# TRAF6 regulates tumour metastasis through EMT and CSC phenotypes in head and neck squamous cell carcinoma

**DOI:** 10.1111/jcmm.13439

**Published:** 2017-11-29

**Authors:** Lei Chen, Yi‐Cun Li, Lei Wu, Guang‐Tao Yu, Wen‐Feng Zhang, Cong‐Fa Huang, Zhi‐Jun Sun

**Affiliations:** ^1^ The State Key Laboratory Breeding Base of Basic Science of Stomatology (Hubei‐MOST) & Key Laboratory of Oral Biomedicine Ministry of Education School & Hospital of Stomatology Wuhan University Wuhan China; ^2^ Department of Oral Maxillofacial‐Head Neck Oncology School and Hospital of Stomatology Wuhan University Wuhan China

**Keywords:** SCCHN, TRAF6, invasion, EMT, cancer stem cells

## Abstract

Epithelial–mesenchymal transition (EMT) is associated with metastasis formation, generation and maintenance of cancer stem cells (CSCs). However, the regulatory mechanisms of CSCs have not been clarified. This study aims to investigate the role of TNF receptor‐associated factor 6 (TRAF6) on EMT and CSC regulation in squamous cell carcinoma of head and neck (SCCHN). We found TRAF6 was overexpressed in human SCCHN tissues, and high TRAF6 expression was associated with lymphatic metastasis and resulted in poor prognosis in patients with SCCHN. In addition, elevated TRAF6 expression was observed in several HNSCC cell lines, and wound healing and transwell assay results showed that TRAF6 knockdown inhibited the migration and invasion ability of the SCCHN cells. Moreover, the expression of Vimentin, Slug and N‐cadherin was down‐regulated and that of E‐cadherin was elevated after TRAF6 knockdown but decreased by transforming growth factor beta 1 (TGF‐β1) and CAL27 similar to mesenchymal cells formed after TGF‐β1 induction. In addition, the expression levels of CD44, ALDH1, KLF4 and SOX2 were inhibited after TRAF6 knockdown, and the anchor‐dependent colony formation number and sphere number were remarkably reduced. Flow cytometry showed TRAF6 knockdown reduced ALDH1‐positive cancer stem cells. We also demonstrated that TRAF6 is closely associated with EMT process and cancer stem cells using a *Tgfbr1*/*Pten* 2cKO mice SCCHN model and human SCCHN tissue microarray. Our findings indicate that TRAF6 plays a role in EMT phenotypes, the generation and maintenance of CSCs in SCCHN, suggesting that TRAF6 is a potential therapeutic target for SCCHN.

## Introduction

Originating from the oral cavity, larynx, hypo‐pharynx and oropharynx, squamous cell carcinoma of head and neck (SCCHN) is the sixth most prevalent cancer worldwide 1. The risk of SCCHN is increased by smoking, alcohol use, betel quid consumption and human papillomavirus infections 2, 3. Recently, the incidence rates of SCCHN have been rapidly increasing in China 4. Despite significant advancement in therapy, no significant improvement in the 5‐year survival rate over the past years has been observed 5, 6. The reasons are that SCCHN has frequent metastasis, high recurrence and poor prognosis 7. Thus, exploring SCCHN pathogenesis and seeking effective treatments are urgently needed to eliminate its threat to the public health.

EMT is a developmental process underlying the acquisition of mesenchymal properties by epithelial cells 8. Currently, EMT is considered an essential step in cancer progression and metastasis, because it enables cancer cells to migrate, invade the surrounding tissues and escape into the blood stream, subsequently leading to the metastasis of primary tumours in other organs 9. Transcriptional regulation by EMT‐inducing transcription factors, such as members of the ZEB, SNAIL and TWIST families, is generally considered the main step in this process 10. Vimentin and N‐cadherin are specific mesenchymal cell markers, and E‐cadherin is a specific epithelial cell marker; meanwhile, Slug belongs to the Snail family and has been proven to be a critical regulator of the EMT process 11, 12. The activation of EMT programme in non‐CSCs enables their conversion into CSCs 10, 12, which exhibit self‐renewal and expanding capability within tumours. These properties contribute to tumour recurrence and metastasis 13. To date, aldehyde dehydrogenase 1 (ALDH1) and CD44 have been reported as promising CSC‐specific markers in SCCHN 14. Transcription factors SOX2 and KLF4 were required for stemness maintenance of CSCs in many cancers 15, 16, 17. However, little is known about the regulatory mechanisms of EMT and CSCs in SCCHN.

TRAF6 is a kind of adaptor protein and E3 ubiquitin ligase that belongs to the family of tumour necrosis factor receptor‐associated factors (TRAFs) 18, 19. TRAF6 is unique because it can activate multiple signalling pathways, such as NF‐κB signalling 19, 20, 21. Recent studies have shown that TRAF6 overexpression occurs in many solid cancers, such as glioma 22, melanomas 23 and oral squamous cell carcinoma 24. Moreover, TRAF6 is an amplified oncogene in human lung cancer 25 and promotes tumour angiogenesis by up‐regulating HIF‐1α 26. TRAF6 knockdown can significantly decrease invasion and metastasis abilities in melanomas and lung cancer 23, 27. Recent studies have shown that high TRAF6 expression is associated with a poor prognosis in glioma and colon cancer 28, 29. TGFβ1 stimulation induces prostate tumour cell scattering and increases the expression levels of Snail and N‐cadherin through the TRAF6‐mediated activation of Rac1/Pak1 pathway 30. A previous study suggested that TRAF6 mediates angiotensin‐II‐induced differential responses in c‐kit(+) cardiac stem cells *via* the non‐canonical TGF‐β signalling pathway 31. TRAF6 can regulate satellite stem cell self‐renewal and function during regenerative myogenesis 32. Therefore, a possible link between EMT‐like CSCs and TRAF6 has been suggested. In addition, TRAF6 not only plays a role in tumorigenesis, but also in the immune system 33, 34. However, the roles of TRAF6 in EMT and CSC regulation in SCCHN remain uncertain.

In this study, TRAF6 was overexpressed in human SCCHN tissues, SCCHN cell line and *Tgfbr1/Pten* 2cKO mice SCCHN model, and TRAF6 was correlated with EMT and CSC markers. Furthermore, TRAF6 knockdown can significantly decrease EMT and stemness in SCCHN cell lines. In addition, increased TRAF6 expression is closely associated with lymph node metastasis in patients with SCCHN, and log‐rank analysis showed that high TRAF6 expression in the overall survival of patients with SCCHN represents poor prognosis.

## Materials and methods

### SCCHN tissue microarrays

For all studies, informed consent was obtained from patients and approved by the School and Hospital of Stomatology of Wuhan University Medical Ethics Committee. All SCCHN specimens were obtained from January 2008 to August 2014 in the Department of Oral and Maxillofacial Surgery, School and Hospital of Stomatology Wuhan University. The clinical stages of SCCHN were classified according to the guidelines of the International Union Against Cancer (UICC 2002), and histological grading was determined according to the scheme of the World Health Organization. These SCCHN tissue arrays of formalin‐fixed were constructed with 1.5 mm core and included 64 confirmed cases of SCCHN (22 cases with lymph node metastasis, Table S1), 38 normal oral mucosa and 12 oral epithelial dysplasia (Dys).

### Antibodies

The following antibodies were used in this study: rabbit monoclonal anti‐TRAF6, anti‐CD44, anti‐SOX2 and anti‐C4.4A (Abcam, Cambridge, UK); mouse monoclonal anti‐CD44 (Abcam); rabbit monoclonal anti‐CD44, anti‐KLF4 and anti‐SOX2 (Epitomics, Burlingame, CA, USA); rabbit polyclonal anti‐ALDH1 (GeneTex Inc., Irvine, CA, USA); rabbit monoclonal anti‐Vimentin, anti‐N‐cadherin, anti‐E‐cadherin, anti‐Slug, anti‐NF‐κB p65, anti‐Phospho‐NF‐κB p65 and anti‐ALDH1 (Cell Signaling Technology, Boston, MA, USA); rabbit polyclonal anti‐AGR2 (Cell Signaling Technology).

### Immunohistochemistry

Paraffin‐embedded patient and mouse tissue were cut into 4‐μm sections and dried at 60°C for 2 hrs. The slides were deparaffnized and rehydrated orderly. For antigen retrieval, the sections were then boiled in 1 mM EDTA buffer solution (pH 8.0) or 0.01 M citric acid buffer solution (pH 6.0) for 5 min. at high pressure. Approximately 3% H_2_0_2_ was incubated at 37°C for 20 min. to quench endogenous peroxidase activity, and 10% normal goat serum (ZSGB‐BIO, Beijing, China) was used to block non‐specific binding. Next, sections were incubated overnight at 4°C with the antibodies mentioned above at the appropriate dilutions, after incubating with corresponding secondary biotinylated immunoglobulin G antibody solution and an avidin‐biotin‐peroxidase reagent, section stained with DAB kit (Mxb Bio) then lightly counterstained with Mayer's haematoxylin (Invitrogen, Carlsbad, CA, USA). The same procedure was performed for the isotype control (IgG, Fig S3).

### Cell culture

SCC9, SCC15 and SCC25 were maintained in DMEM/F12 with 10% foetal bovine serum and 400 ng/ml hydrocortisone and CAL27 were maintained in DMEM/high glucose with 10% foetal, all those SCCHN cell lines were bovine at 5% CO2 and 37°C in a humidified incubator and purchased from the American Type Culture Collection (ATCC, Manassas, VA, USA). Primary cultured oral keratinocyte cell line (OKC, 2–3 passive) was cultured in a defined keratinocyte serum‐free medium (KSFM; GIBCO BRL, Carlsbad, CA, USA) 35.

### TRAF6 siRNA and transfection

Cells were seeded in six‐well plates (NEST Biotechnology Co.LTD., Wuxi, China) at a density of 1 × 10^5 ^cells/well and replaced with serum‐free medium when density reached about 80%. 10–20 ul TRAF6 siRNA or negative control siRNA (GenePharma, Shang Hai, China) was mixed with 125 μl opti‐MEM medium (Thermo Fisher Scientific, Waltham, MA, USA). 3.75 μl Lipofectamine‐3000 (Thermo Fisher Scientific) was added to another 125 μl opti‐MEM medium and fully mixed. This was gently mixed and then incubated for 5 min. at room temperature. The untreated groups (without siRNA and transfection agent) were used as blank controls. The mixture was then added to the six‐well plates. Cells were incubated at 37°C for 2–4 days. The transfected cells were then analysed.

### Wound healing and transwell assay

For the wound healing assay, transfected cells and control cells were re‐suspended in serum‐free medium, then seeded in six‐well plates at a density of 10^6^/well. An artificial scratching was made using a 20 μl pipette tip when density reached about 95%. After washing three times with PBS, the medium was replaced with fresh serum‐free medium. Images were captured by inverted microscope (Nikon, Japan) at 0 and 48 hrs. The wound healing rate was analysed by Image J 36. For transwell assay, 8‐μm‐pore chamber in 24‐well plates (Corning, Corning, NY, USA) was prepared with 40 ul matrigel (BD, diluted (1:3) in serum‐free DMEM) and placed at room temperature overnight to solidify completely. Transfected cells and control cells were re‐suspended in serum‐free medium, then seeded in the upper chamber at a density of 1 × 10^5^/100 μl, and 600 μl medium containing 10% FBS was added to the lower room. After 48 hrs incubation, cells were fixed with 4% paraformaldehyde and stained with crystal violet, and uninvaded upper cells were removed. Invaded cells images were captured at 20× magnification. Invaded cell numbers were analysed by Image J. Each assay was performed in triplicate.

### Sphere‐forming assay and colony formation assay

For sphere‐forming assay, single‐cell suspensions were re‐suspended in culture medium with 1% N2 supplement (Gibco, Carlsbad, CA, USA), 10 ng/ml bFGF (Invitrogen) and 10 ng/ml EGF (Gibco) according to the manufacturer's instructions and plated in ultra‐low attachment plates (Corning) at a density of 1 × 10^3^ per well. Inverted microscope and 96‐well plates (NEST Biotechnology Co.LTD.) were used to count the tumour sphere number. To obtain re‐adherent cells, spheres were digested and re‐suspended in DMEM/high glucose with 10% foetal medium and plated in six‐well plates (NEST Biotechnology Co. LTD.). Protein extraction was performed after 72 hrs. For colony formation assay, cells were seeded in 12‐well plates at a density of 200 cells/well with 10% FBS contained‐medium. After 7 days incubation (10 days for SCC25), the colonies were fixed with 4% paraformaldehyde and stained with crystal violet. The numbers of colonies were counted. Each assay was performed in triplicate.

### Flow cytometry

Single‐cell suspensions were adjusted to a concentration of 1 × 10^6^/ml in ALDEFLUOR™ Assay Buffer (STEMCELL Technologies, Vancouver, BC, Canada). Then cells were incubated with ALDEFLUOR™ Reagent for 30 min. at 37°C according to the manufacturer's instructions. Cells were analysed using a Flow cytometer (CytoFLEX, BECKMAN COULTER Brea, CA, USA).

### TGF‐β1‐induced EMT assay

EMT can be induced by many extracellular ligands, and TGF‐β and TGF‐β‐related extracellular ligands have emerged as major inducers of EMT in development and cancer 37. Transfected cells and control cells were re‐suspended in serum‐free medium then seeded in 6‐well plates at a density of 10^6^/well. After 12‐hr incubation, cells were treated with 10 ng/ml recombinant human TGF‐β1 (PEPROTECH, Rocky Hill, NJ, USA) for 48 hrs. Images of cell morphology or immunofluorescence were taken then.

### NF‐κB activation assay

Compared to other members, TRAF6 is unique because it can activate multiple signalling pathways including NF‐κB signalling 19, 20, 21. Transfected cells and control cells were re‐suspended in serum‐free medium then seeded in six‐well plates at a density of 10^6^/well. After 24‐hr incubation, cells were treated with 20 ng/ml recombinant human TNF‐α (PEPROTECH) for 30 min. or 48 hrs. Protein extraction or immunofluorescence was performed then.

### Knock out SCCHN mouse model

Time inducible and tissue‐specific *Tgfbr1/Pten* 2cKO mice (*K14‐Cre* ^ERtam^; *Tgfbr1* ^flox/flox^; *Pten* ^flox/flox^), *Tgfbr1* cKO mice (*K14‐Cre* ^ERtam^; *Tgfbr1* ^flox/flox^), *Pten* cKO mice (K14‐Cre^ERtam^; *Pten* ^flox/flox^) were maintained and genotyped according to published protocols 38. All the mice were maintained in FVBN/CD1/129/C57 mixed background. All animal studies were approved and supervised by the Animal Care and Use Committee of Wuhan University.

### Cell immunofluorescence

CAL27 cells were seeded on a cover glass slide chamber (Millipore, Billerica, MA, USA). Transfection cells were fixed with 4% paraformaldehyde at room temperature for 15 min. and then treated with 0.5% triton X‐100. After blocking with 2.5% BSA for 1 hr, the cells were incubated with primary antibody mentioned above overnight at 4°C. Cells were then incubated with secondary fluorescent antibodies (DyLight 488 anti‐rabbit, DyLight 594 anti‐rabbit and antimouse; Thermo Scientific, USA) with DAPI (Jackson ImmunoResearch Laboratories, Inc, West Grove, PA, USA) for 1 hr in the dark at room temperature. The slides were observed by a confocal laser scanning microscope (FV300, Olympus Life Science, Tokyo, Japan).

### Western blotting

The Western blotting analysis was conducted as previously described 39. Briefly, cultured cells and mice tissue were lysed using M‐PER or RIPA reagent (Pierce, Rockford, IL, USA) containing a complete mini protease inhibitor cocktail and phosphate inhibitors (Roche, Branchburg, NJ, USA). After denaturation, the total protein was separated using 10% SDS–polyacrylamide gel electrophoresis and transferred onto polyvinylidene fluoride membranes (Millipore). After blocking with 5% non‐fat dry milk at room temperature for 1 hr, the blots were incubated overnight with the corresponding primary antibodies at dilutions recommended by the suppliers at 4°C and by incubation with horseradish peroxidase‐conjugated secondary antibody (Pierce) for 1 hr at room temperature. Finally, the blots were detected with ECL kit (Advansta, Menlo Park, CA, USA). GAPDH was detected on the same membrane and used as a loading control.

### Scoring system, hierarchical clustering and data visualization

All slides were scanned using an Aperio Scanscope CS (Aperio, San Diego, CA, USA) with background subtraction and quantified for pixel quantification by Aperio Quantification software (Version 9.1). Scoring system, hierarchical clustering and data visualization were performed according to our previous studies 38, 39. Histoscore of pixel quantification was calculated according to the formula (3+) × 3+ (2+) × 2+ (1+) × 1 as previously described 40. The results were presented by the Cluster 3.0 and Java TreeView 1.5.5. Clustered data and tissue samples were arranged on the horizontal axis and vertical axis, respectively. Closely related biomarkers were placed tightly together.

### Statistical analysis

Statistical data analysis was performed with GraphPad Prism 7 statistical packages for Windows (GraphPad Software, Inc., La Jolla, CA, USA). Paired/unpaired *t*‐test was adopted for data between two experimental groups, and a one‐way ANOVA test was used in multiple group's analysis. Two‐tailed Pearson's statistics was utilized for correlation analysis based on confirmation of the sample with Gaussian distribution. Overall survival curves were estimated by the Kaplan–Meier method and compared by the log‐rank test. The cut‐off was confirmed by an online software: Cutoff Finder 41. Data were presented as mean ± S.E.M.; statistical significance was defined as the *P*‐value was <0.05. *P <* *0.05; **P <* *0.01; ***P <* *0.001; ns, not significant.

## Results

### Elevated TRAF6 in human SCCHN

**Figure 1:**
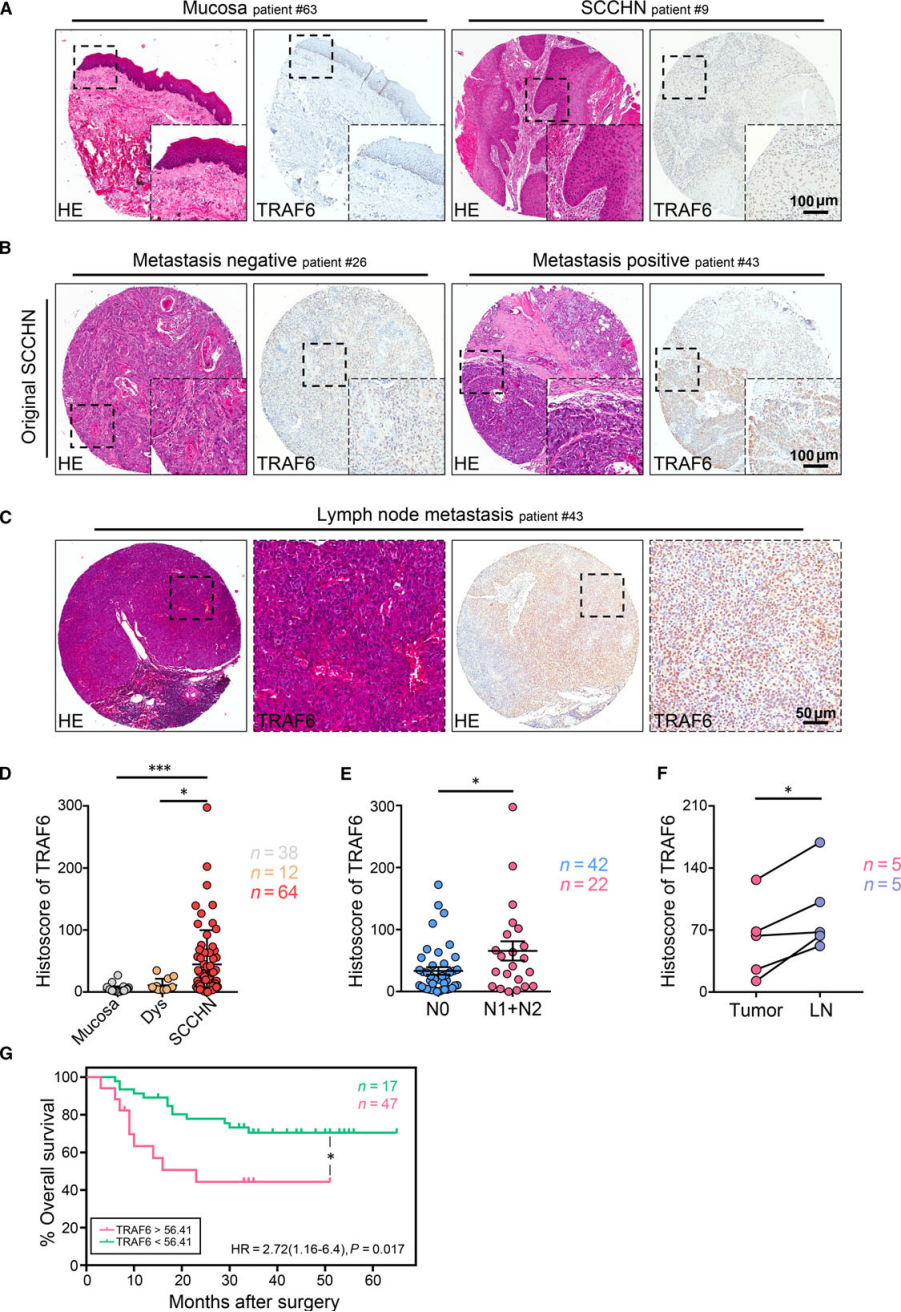
Elevated expression of TRAF6 was closely related to lymph node status in human SCCHN tissue. (**A**) Representative haematoxylin and eosin (HE) and immunohistochemistry (IHC) staining of TRAF6 in normal oral mucosa (left) and SCCHN (right). Scale bars = 100 μm. (**B**) Representative HE and IHC staining of TRAF6 in metastasis‐free SCCHN (left) and SCCHN with lymph node metastasis (right). Scale bars = 100 μm. (**C**) Representative HE and IHC images of TRAF6 in metastatic lymph node. Scale bars = 50μm. (**D**) Quantification of IHC histoscore of TRAF6 among oral mucosa (*n* = 38), dysplasia (Dys, *n* = 12) and head and neck squamous cell carcinoma (SCCHN,* n* = 64). One‐way ANOVA with post‐Tukey test. *P <* *0.05; ***P <* *0.001. (**E** and **F**) Quantification of IHC histoscore of TRAF6 showed the expression of TRAF6 in SCCHN with lymph node metastasis (N1 + N2, *n* = 22) was significantly higher than in metastasis‐free SCCHN (N0, *n* = 42) (*t*‐test, *P <* *0.05) and was remarkably increased in metastatic lymph nodes than in primary tumour (*n* = 5, paired *t*‐test, *P <* *0.05). (**G**) Kaplan–Meier survival curve of TRAF6 showed the overall survival of patients with high‐TRAF6 expression (*n* = 47) was lower than low‐TRAF6 expressions patients (*n* = 17) (*P *<* *0.05). All data are presented as mean ± S.E.M.

To investigate the role of TRAF6 in SCCHN, we analysed *TRAF6* mRNA expression level from the publicly available cancer microarray database Oncomine^®^. We found that *TRAF6* mRNA expression was increased in SCCHN compared with normal counterpart (*P *=* *2.25E‐4, Fig. S1A). Furthermore, TRAF6 protein expression was detected using immunohistochemistry in our SCCHN tissue microarray (Fig. 1A). The results show that positive TRAF6 staining was mainly located in the cytoplasm of cancer cells and partially in the nucleus, and its expression was significantly increased in SCCHN (*n* = 64) versus dysplasia (*n* = 12, *P *<* *0.05) and normal mucosa tissues (*n* = 38, *P *<* *0.001, Fig. 1A and D). We then analysed the relationship between TRAF6 and clinicopathological features. The analysis results indicated that the original SCCHN with lymph node metastasis status (N1 + N2, *n* = 42, *P *<* *0.05) had stronger immunoreactivity than the original SCCHN with lymph node‐negative status (N0, *n* = 22, Fig. 1B and E), and the expression of TRAF6 was still located in the cytoplasm and partially in the nucleus of cancer cells. Additionally, TRAF6 expression was increased in metastasis lymph node (*n* = 5, *P *<* *0.05, Fig. 1C and F), but it was higher in the nucleus than that in the cytoplasm. However, elevated TRAF6 expression was not significantly correlated with pathological grades (I–III) and tumour size (T1–T4) (Fig. S1C). In addition, previous studies reported that patients with high TRAF6 expression have a worse survival rate in glioma and colon cancers 28, 29. Therefore, we performed the Kaplan–Meier method to explore the prognostic value of TRAF6 in SCCHN. As expected, log‐rank analysis showed that the overall survival rate of patients with high TRAF6 expression levels presented poor prognosis *(P *=* *0.017, Fig. 1G). Based on these results, we speculated that TRAF6 may play a role in SCCHN progression, especially in metastasis, which is a characteristic of cancer stem cells.

### TRAF6 knockdown **affects the** EMT process **in SCCHN cells**

**Figure 2:**
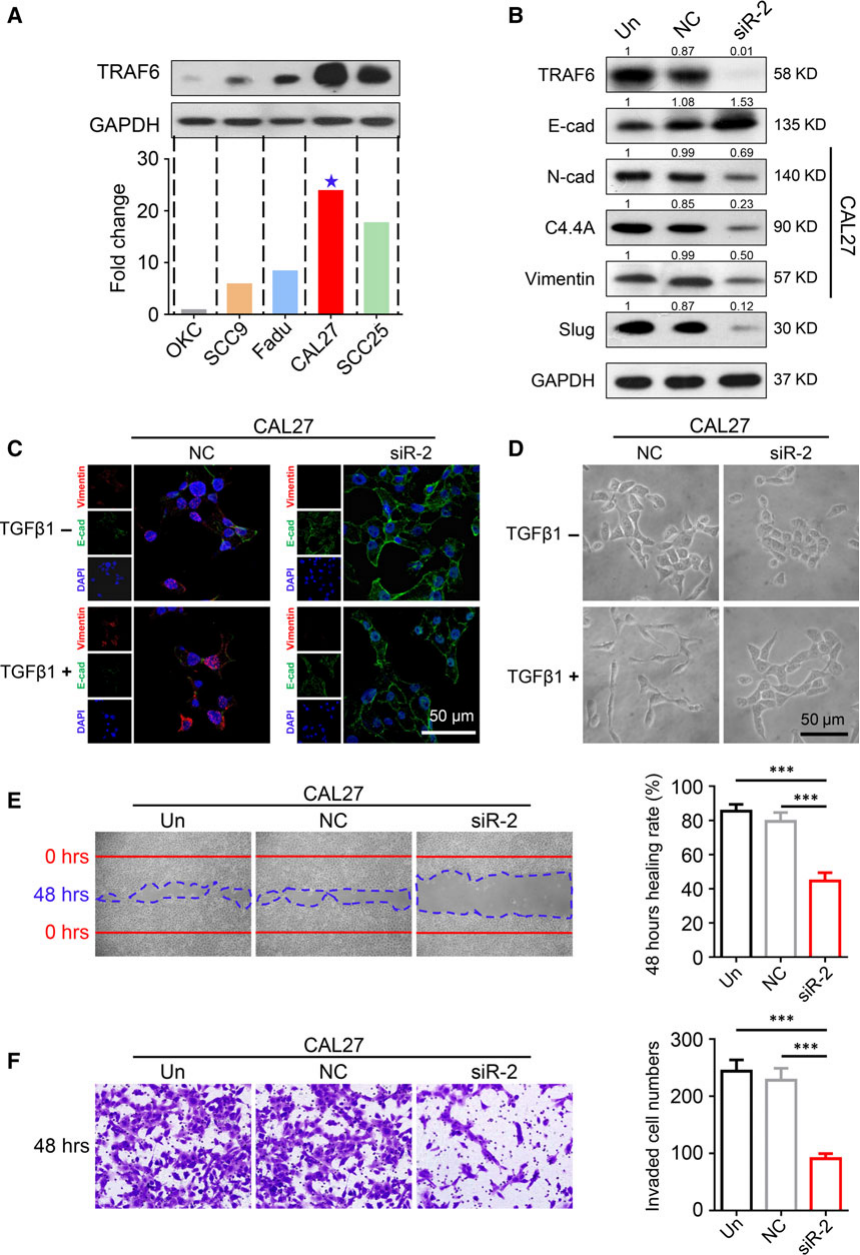
EMT process, invasiveness and metastasis ability were inhibited by knocking down TRAF6 in SCCHN cells. (**A**) Western blot analysis was performed to examine TRAF6 expression in several SCCHN cell lines and OKC cell line. (**B**) Western blot results showed an obviously drop in mesenchymal‐related proteins N‐Cadherin, Vimentin, Slug and a slightly raise in epithelial‐related protein E‐Cadherin after TRAF6 siRNA transfecting in CAL27 cell lines and C4.4A also reduced. (**C**) Representative immunofluorescence images of TGFβ1‐induced EMT assay (48 hrs) in TARF6 knockdown and control groups. Vimentin (red), E‐Cadherin (green) and DAPI (blue); Scale bar = 50 μm. (**D**) Representative photographs of CAL27 cell morphologies after TGFβ1 treating (10 ng/ml, 48 hrs) in TARF6 knockdown and control groups. Scale bar = 50 μm. (**E**) Representative microphotographs of wounding healing assay (magnification, 20×) and quantitative statistics of healing rate. Photograph was taken at 0 and 48 hrs after scratching (Mean ± S.E.M., ***P <* *0.001). (**F**) Representative microphotographs of transwell assay (magnification, 40×) and quantitative statistic of invaded cells. Photograph was taken at 48 hrs after cell plating (Mean ± S.E.M., ***P <* *0.001).

To investigate further the function of TRAF6 in SCCHN, we also examined TRAF6 expression level in several SCCHN cell lines, and oral keratinocyte (OKC) was used as a control. TRAF6 was highly expressed in SCC9, SCC15, SCC25 and CAL27 cell lines, among which the CAL27 cells had the highest expression level (Fig. 2A). CAL27 and SCC25 were then selected for subsequent *in vitro* functional assay. The results showed that TRAF6 siRNA can effectively reduce TRAF6 protein expression (Fig. 2B). Interestingly, EMT‐related proteins N‐cadherin, Vimentin and Slug were significantly reduced after transfecting with TRAF6 siRNA (Figs 2B and S2E).

EMT can be induced by many extracellular ligands, and TGF‐β and TGF‐β‐related extracellular ligands have emerged as major inducers of EMT in development and cancer 37. To study further the relationship between TRAF6 and EMT process in SCCHN, TGF‐β1‐induced EMT assay was carried out. Our immunofluorescence results confirmed that E‐cadherin expression was up‐regulated and Vimentin was down‐regulated in the siRNA group. In addition, treating with recombinant human TGF‐β1 decreased the expression of E‐cadherin and increased Vimentin expression in the negative control group, but there were no obvious changes detected in the siTRAF6 group (Fig. 2C). Additionally, cells formed like mesenchyme (spindle‐like morphology) after TGF‐β1 induction were not observed in the siTRAF6 group (Fig. 2D). Therefore, TRAF6 may be an important element in the process of EMT in SCCHN.

In addition, given that the expression of TRAF6 is associated with lymph node metastasis in human SCCHN, we hypothesized that TRAF6 may also play a role in the migration and invasion of SCCHN cell lines. For hypothesis testing, wound healing and transwell assays were performed with the most efficient siRNA sequence in the CAL27 cells. Forty eight hours after artificial scratching, TRAF6 knockdown significantly reduced the healing capacity to a greater extent than that of the control group (*P *<* *0.001, Fig. 2E). In transwell invasion assay, 48 hrs after cell plating, more invasion cells were observed in blank control and negative control counterpart in comparison with the TRAF6 knockdown group (*P *<* *0.001, Fig. 2F). These results were also confirmed in SCC25 cell line (Fig. S2A and B). Overall, TRAF6 knockdown effectively reduces the invasion ability and the EMT process in human SCCHN cells *in vitro*.

### TRAF6 is related to CSCs phenotype in SCCHN cells

**Figure 3:**
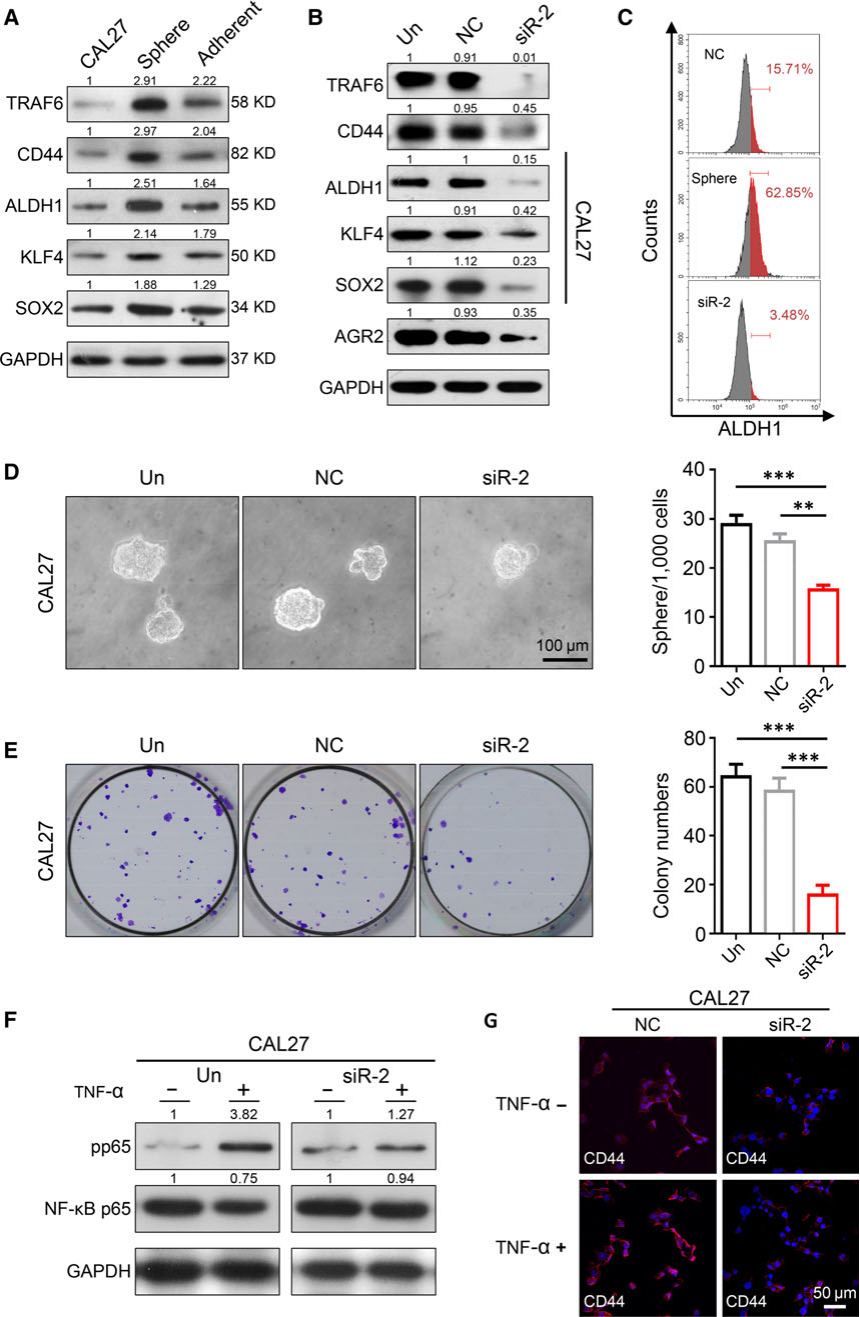
Effect of TRAF6 knockdown on cancer stem cells formation in SCCHN cell lines. (**A**) Western blot analysis showed that TRAF6, CD44, ALDH1, KLF4 and SOX2 proteins expression was raised in sphere compared to CAL27 cells but was reduced in re‐adherent cells compared to sphere. (**B**) Western blot analysis showed that CD44, ALDH1, KLF4, SOX2 and AGR2 proteins expression were remarkably decreased after knocking down TRAF6 in CAL27 cell line. (**C**) Flow cytometry dot plots for ALDH1^+^ cell in negative control (15.71%), sphere (62.85%) and si‐TRAF6 CAL27 groups (3.48%). (**D**) Representative microphotographs of sphere formation and quantitative analysis. Scale bars = 100 μm. (Mean ± S.E.M., **P <* *0.01, ***P <* *0.001). (**E**) Representative images of anchor‐dependent colony formation and quantitative analysis. (Mean ± S.E.M., ***P <* *0.001). (**F**) Western blot analysis showed that knocking down TRAF6 blocked the activation of NF‐κB pathway in CAL27 cell line after treating with 20 ng/ml recombinant human TNF‐α for 48 hrs. (**G**) Representative immunofluorescence images of CD44 (red) and DAPI (blue) in NF‐κB activation assay (20 ng/ml TNF‐α, 48 hrs); Scale bar = 50 μm.

Sphere‐formation assay is a widely used method for detecting cancer stem cell characteristics, and the higher the sphere numbers are, the stronger stemness cells are. Additionally, spheres possess stronger stemness property than progenitor cells, but spheres would begin to attach onto its culture vessel when cultured with conventional 10% serum‐containing medium, and accompanied by a decrease in stemness ability (Fig. 3A). Furthermore, TRAF6 expression was consistent with the changes in the CSC‐associated proteins (CD44, ALDH1, KLF4 and SOX2), as indicated by the Western blot analysis results (Fig. 3A). To study further the relationship between TRAF6 and CSCs, we tested CSC‐related protein change after TRAF6 knockdown. Results showed that CSC markers CD44, ALDH1, KLF4, SOX2 and AGR2 are down‐regulated after reducing TRAF6 expression (Figs 3B and S2E). The results of flow cytometry also showed that knockdown of TRAF6 reduced the number of ALDH1‐positive CSCs (Fig. 3C).Then sphere‐formation assay was performed to verify the potential role of TRAF6 in the stemness ability of CAL27 cells. Notably, TRAF6 knockdown obviously reduced the number of spheres when compared with the mock group and negative control (*P *<* *0.001, Fig. 3D). Moreover, TRAF6 knockdown can also reduce anchor‐dependent colony formation number (*P *<* *0.001, Fig. 3E). We also found TRAF6 siRNA could reduce sphere and colony formation number in SCC25 (Fig. S2C and D).

Furthermore, TRAF6 is unique because it can activate multiple signalling pathways including NF‐κB signalling 19, 20, 21. Recently, reports showed that NF‐κB regulation of critical target genes, prominently including cytokines and EMT transcription factors, drive CSC phenotypes 42. Our Western blot results show that TNF‐α stimulation can significantly increase the expression of phospho‐NF‐κB, but this process seemed to be partially inhibited after TRAF6 knockdown (Fig. 3F). In addition, by immunofluorescence experiments, we also found that the expression of CD44 was significantly increased after TNF‐α stimulation in CAL27, but this change was not prominent in the TRAF6 knockdown group (Fig. 3G). Together, these data indicate that TRAF6 may play a key role in the CSC regulation through NF‐κB signalling in human SCCHN cells, and the specific mechanisms need further researches.

### TRAF6 was correlated with EMT and CSCs in mice and human SCCHN

**Figure 4:**
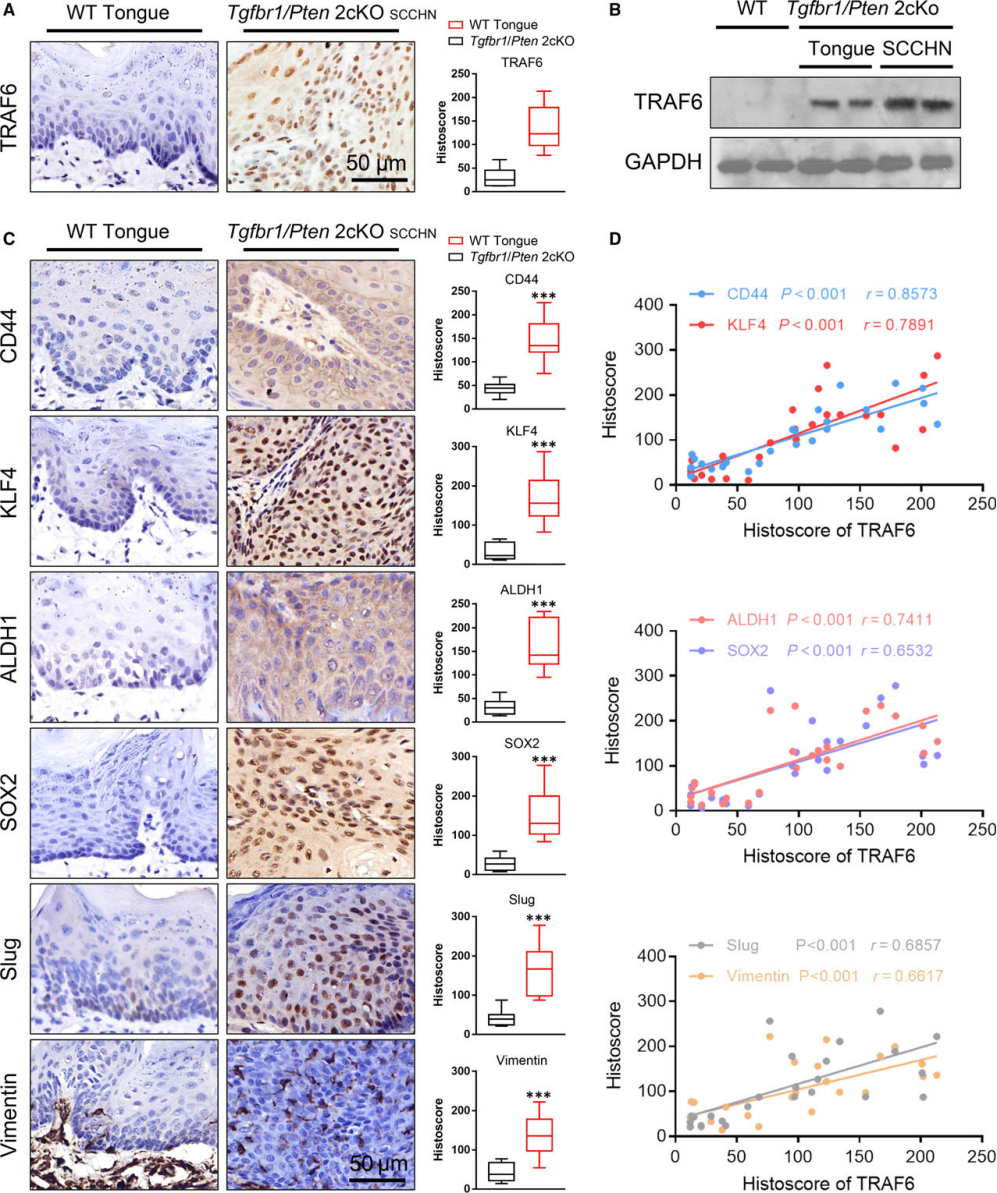
High expression of TRAF6 is correlated with CSCs and EMT‐related protein in *Tgfbr1*/*Pten* 2cKO mice. (**A**) Representative IHC staining and quantification of TRAF6 in wild‐type mice (WT) tongue and *Tgfbr1*/*Pten* 2cKO mice tumour (SCCHN), (*t*‐test, Mean ± S.E.M., ***P <* *0.001); Scale bars = 50 μm and (**B**) Western blot analysis of TRAF6 expression in these tissues. (**C**) Representative IHC staining and quantification of CD44, KLF4, ALDH1, SOX2, Slug and Vimentin in wild‐type mice (WT) tongue and *Tgfbr1*/*Pten* 2cKO mice tumour (SCCHN), (*t*‐test, Mean ± S.E.M., ***P <* *0.001); Scale bars = 50 μm. (**D**) Correlation of TRAF6 with CD44 (*P *<* *0.001, *r* = 0.8573), KLF4 (*P *<* *0.001, *r* = 0.7891), ALDH1 (*P *<* *0.001, *r* = 0.7411), SOX2 (*P *<* *0.001, *r* = 0.6532), Slug (*P *<* *0.001, *r* = 0.6857) and Vimentin (*P *<* *0.001, *r* = 0.6617) in mice SCCHN (10 wild‐type mice tongues and 15 mice SCCHN, a dot represents a sample).

EMT and CSCs are known to play important roles in cancer recurrence and metastasis 43. To explore whether an association is present between TRAF6 with EMT and cancer stem cells, we first inquired the Tissue Cancer Genome Atlas dataset (TCGA). Cluster analysis about mRNA expression between *TRAF6* and some common EMT and CSC markers has been operated; result showed that *TRAF6* and EMT and CSC mRNAs in SCCHN are correlated (Fig. S1B), especially a strong correlation with *CD44*. *Tgfbr1* and *Pten* knockout mice with *de novo* SCCHN tumorigenesis have been reported in our previous study 38. To determine whether TRAF6 expression was also associated with CSCs in the mouse model, immunohistochemistry and Western blot were performed. The results showed that the expression of TRAF6 in SCCHN mouse model was significantly increased (Fig. 4A and B). Apart from this, the expression level of CSCs‐related proteins:CD44, ALDH1, KLF4, SOX2 and EMT‐related proteins: Slug, Vimentin were also remarkably elevated in mice SCCHN tumour than that in wild‐type normal tongue tissue (Fig. 4C). Furthermore, we found that the protein expression of TRAF6 was statistically associated with those CSC and EMT markers in mice SCCHN tissues (CD44, *P *<* *0.001, *r* = 0.8573; KLF4, *P *<* *0.001, *r* = 0.7891; ALDH1, *P *<* *0.001, *r* = 0.7411; SOX2, *P *<* *0.001, *r* = 0.6532; Slug, *P *<* *0.001, *r* = 0.6857; Vimentin, *P *<* *0.001, *r* = 0.6617; Fig. 4D) by Spearman rank correlation coefficient test and linear tendency test.

**Figure 5:**
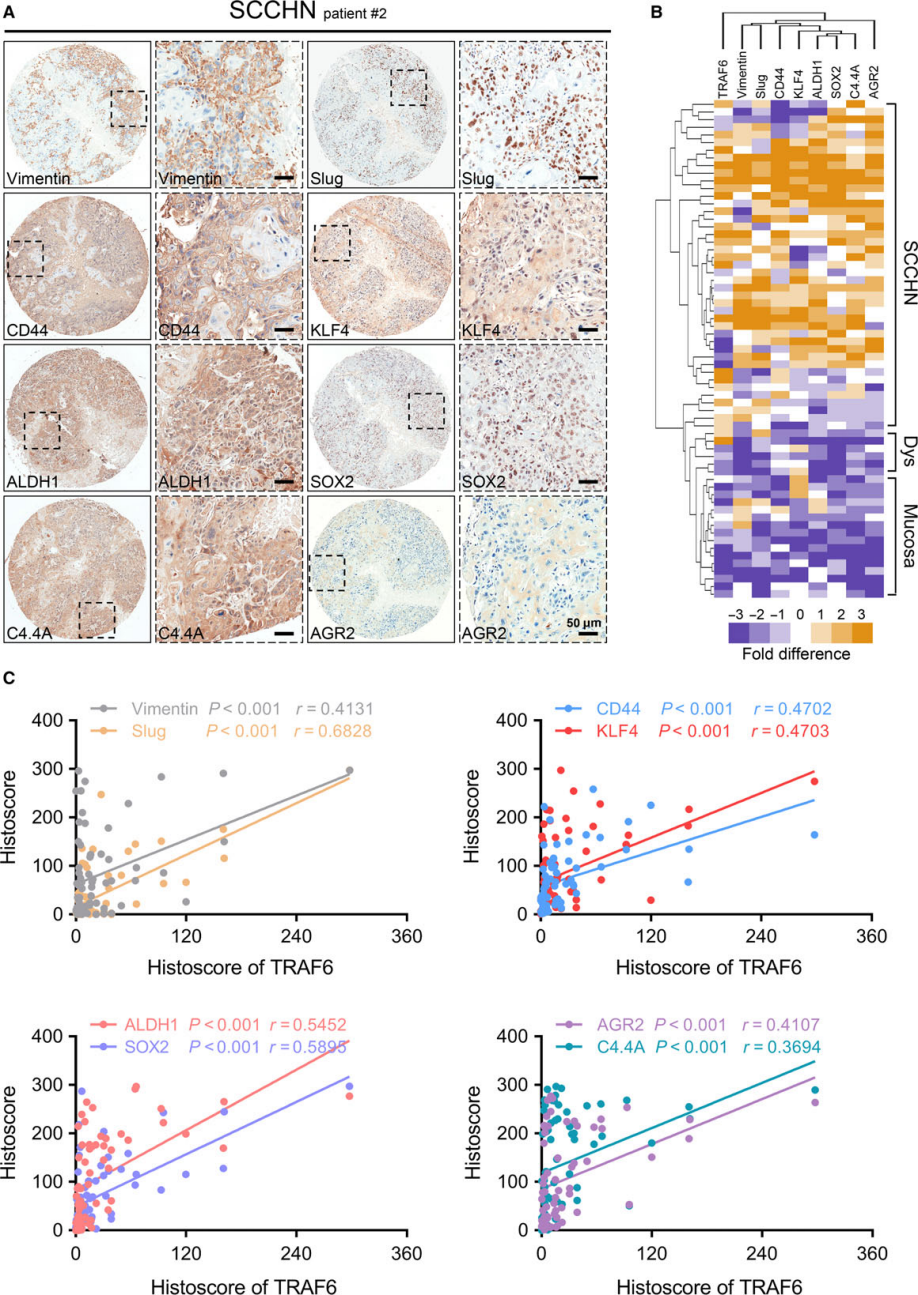
Increased TRAF6 expression is associated with EMT and cancer stem cells markers in human SCCHN tissue. (**A**) Representative IHC staining of Vimentin, Slug, CD44, KLF4, ALDH1, SOX2, C4.4A and AGR2 in human SCCHN tissue. Scale bars = 50 μm. (**B**) Hierarchical clustering of TRAF6, Vimentin, Slug, CD44, KLF4, ALDH1, SOX2, C4.4A and AGR2 in human SCCHN tissue microarray. IHC staining was clustered with cluster and visualized with Java Treeview. (**C**) Pearson's correlation coefficient test of TRAF6 with EMT and CSCs‐related proteins (Vimentin, *P *<* *0.001, *r* = 0.4131; Slug, *P *<* *0.001, *r* = 0.6828; CD44, *P *<* *0.001, *r* = 0.4702; KLF4, *P *<* *0.001, *r* = 0.4703; ALDH1, *P *<* *0.001, *r* = 0.5452; SOX2, *P *<* *0.001, *r* = 0.5895; C4.4A, *P *<* *0.01, *r* = 0.3694; AGR2, *P *<* *0.001, *r* = 0.4107; respectively). The data are presented as dot plots of each specimen, with statistics including mucosa (*n* = 38), dysplasia (*n* = 12) and primary SCCHN (*n* = 64).

Moreover, EMT markers (*i.e*. Vimentin and Slug) and CSC markers (*i.e*. CD44, KLF4, ALDH1 and SOX2) are used to examine the protein expression levels in human SCCHN tissue microarray; results showed that all the proteins mentioned above were overexpressed in SCCHN tissues (Fig. 5A). Additionally, we found that the localization of these proteins was different: Vimentin was expressed in the tumour stroma, AGR2 was located in the cytoplasm of cancer cells, CD44 was mainly located in cancer cells membrane and partially in the cytoplasm, Slug and SOX2 were expressed in the nucleus of tumour cells, but KLF4, ALDH1 and C4.4A were expressed in the cytoplasm and nucleus of cancer cells. Subsequently, we were surprised to discover that the expression of TRAF6 in SCCHN was significantly correlated with EMT markers (Vimentin, *P *<* *0.001, *r* = 0.4131; Slug, *P *<* *0.001, *r* = 0.6828; Fig. 5C) and CSC markers (CD44, *P *<* *0.001, *r* = 0.4702; KLF4, *P *<* *0.001, *r* = 0.4703; ALDH1, *P *<* *0.001, *r* = 0.5452; SOX2, *P *<* *0.001, *r* = 0.5895; Fig. 5C). Apart from this, hierarchy clustering and linear regression analyses showed that expression of EMT and CSC markers were very close to the expression of TRAF6 (Fig. 5B). In addition, our previous data have proved that C4.4A and AGR2 were activated in SCCHN and associated with EMT or CSCs 39, 44; moreover, both of them have a strong correlation and a similar expression pattern with TRAF6 (C4.4A, *P *<* *0.01, *r* = 0.3694; AGR2, *P *<* *0.001, *r* = 0.4107; Fig. 5B and C). All the above suggests that TRAF6 plays a crucial role on EMT process and CSCs regulation in human SCCHN.

## Discussion

Accumulating evidence has shown that CSCs play an important role in chemoresistance, tumorigenesis, recurrence and metastasis in SCCHN 14. Recent studies have shown that the EMT phenotype is associated with the invasive characteristics of CSCs, which possess an EMT phenotype that may predominate in tumour invasion, metastasis and drug resistance 43, 45, 46. During the process of cancer metastasis, CSCs undergo EMT, which is acquiring mesenchymal features, migrating to adjacent stromal tissues and invading blood or lymph vessels 47. Despite all these recent findings, the specific mechanisms between EMT and CSCs in head and neck cancer have not been clearly clarified.

TRAF6 belongs to the TRAF family 18, 19, which has been reported to promote cancer tumorigenesis, invasion and metastasis 22, 23, 48, and participate in a variety of signal pathways 49. However, little is known about its association with SCCHN development. We performed a comprehensive immunohistochemical analysis of TRAF6 expression in SCCHN tumours. In the present study, positive TRAF6 staining was mainly located in the cytoplasm of cancer cells and partially in the nucleus, and its expression was significantly increased in SCCHN versus dysplasia and normal mucosa tissues. Recent studies have shown that TRAF6 overexpression has been observed in solid cancers such as glioma 22, melanomas 23 and oral squamous cell carcinoma 24. Subsequently, we observed a stronger immunoreactivity in SCCHN with lymph node metastasis in comparison with metastasis‐negative SCCHN, and an elevated expression of TRAF6 in metastatic lymph node than in its original tumour. Additionally, some studies have shown that high TRAF6 expression represented a poor prognosis in glioma and colon cancers 28, 29. Therefore, we performed the Kaplan–Meier method to explore the prognostic value of TRAF6 in SCCHN. The overall survival rate of patients with high TRAF6 expression represents a poor prognosis, which was consistent with the researches mentioned above. Furthermore, TRAF6‐conferred poor prognosis is independent of tumour size or pathological grades.

Furthermore, EMT has also been involved in the generation of cancer stem cells, and subpopulation of cells identified within solid tumours exhibit self‐renewal and expanding capability, and contribution to cancer recurrence, metastasis and chemoresistance 12, 50. To investigate the relationships between EMT, CSCs and TRAF6 in head and neck cancer, we discovered that the expression of TRAF6 in SCCHN was significantly correlated with EMT markers (*i.e*. Vimentin and Slug) and CSC markers (*i.e*. CD44, KLF4, ALDH1 and SOX2). Our previous data have proven that C4.4A and AGR2 were activated in SCCHN and associated with EMT or cancer stem cell 39, 44. In this study, both C4.4A and AGR2 have a strong correlation and a similar expression pattern with TRAF6. In addition, hierarchy clustering and linear regression analyses showed that expression of EMT and CSC markers was very close to the expression of TRAF6. Recently, reports showed that NF‐κB regulation of critical target genes, prominently including cytokines and EMT transcription factors, drives CSC phenotypes 42. Moreover, our results showed that TRAF6 participated in the NF‐κB activation, which indicated that TRAF6 may play a key role in the CSC regulation through NF‐κB signalling in human SCCHN cells. In addition, TRAF6 participates in IL‐1β signalling that promotes cancer cell invasion in oral squamous cell carcinoma 24 and tumour angiogenesis by up‐regulated HIF‐1α 36. All the above data suggested that TRAF6 may play a crucial role on EMT process and CSCs regulation in human SCCHN.

Loss of E‐cadherin expression and elevated Vimentin and N‐cadherin expression levels is a fundamental event in EMT 9. Al‐Azayzih observed that stimulation with TGFβ1 induced prostate tumour cell scattering and increased expression of Snail and N‐cadherin through TRAF6‐mediated activation of Rac1/Pak1 pathway 30. TRAF6 knockdown can significantly decrease invasion and metastasis abilities in melanomas and lung cancer 23, 27. In the present study, we found that TRAF6 was highly expressed in SCCHN cell lines. Interestingly, EMT‐related proteins N‐cadherin, Vimentin and Slug were significantly reduced after transfecting with TRAF6 siRNA, and the expression of E‐cadherin was elevated**.** TGF‐β and TGF‐β‐related extracellular ligands have emerged as major inducers of EMT in cancer 37. The results showed E‐cadherin expression was decreased and Vimentin was obviously increased after recombinant human TGF‐β1 treatment and no change was detected in the siTRAF6 group. Moreover, we found that TGF‐β1 induced mesenchymal‐like morphological changes. Therefore, TRAF6 may be an important element in the process of EMT in SCCHN. Recent study showed that TRAF6 promotes TGFβ‐induced invasion in prostate cancer 51, which is consistent with our results. A previous study suggested that TRAF6 mediated angiotensin‐II‐induced differential responses in c‐kit(+) cardiac stem cells *via* the non‐canonical TGF‐β signalling pathway 31. TRAF6 can regulate satellite stem cell self‐renewal and function during regenerative myogenesis 32. Our results showed that CSC markers CD44, ALDH1, KLF4, SOX2 and AGR2 were down‐regulated after reducing TRAF6 expression. The sphere‐formation assay was performed to verify the potential role of TRAF6 in the stemness ability. Notably, TRAF6 knockdown obviously reduced the number of spheres and also reduced anchor‐dependent colony formation number in SCCHN cells. NF‐κB signalling plays a crucial role in cancer stem cell biology 42, and TNF‐α‐induced NF‐κB activation elevated CD44 expression in SCCHN cells, but TRAF6 knockdown markedly attenuated this process. In addition, the results showed that TRAF6 knockdown significantly reduced the healing capacity and invasion ability. Therefore, a possible link between EMT‐like CSCs and TRAF6 has been suggested. These results suggested that TRAF6 can play an important role in EMT phenotypes and CSC generation and maintenance in human SCCHN. It can be a potential new target for therapeutic strategies for SCCHN.

In summary, our results demonstrate that TRAF6 plays a functional role in the EMT phenotypes and in the CSC generation and maintenance in head and neck cancer cells. Therefore, inhibition of TRAF6 may be an effective approach to eliminate CSCs, reversing the EMT phenotype and consequently eradicating cancer cells to improve the prognosis of patients with head and neck cancer.

## Conflict of interest

The authors declare that they have no competing interests.

## Author contributions

C.F.H., L.C. and Z.J.S. performed experiments; C.F.H., W.F.Z., Z.J.S. participated in the design of the study; C.F.H., L.C. and Z.J.S. participated in data analysis and statistical analysis; L.C., C.F.H., Y.C.L., L.W., G.T.Y., W.F.Z. and Z.J.S. drafted and revised the manuscript. All authors read and approved the final manuscript.

## Supporting information


**Figure S1.** Analyses from several online databases and clinicopathological analysis.

**Figure S2.** TRAF6 knockdown reduces the migration, invasion and self‐renewal abilities in SCC25.

**Figure S3.** IgG Isotype controls for IHC experiments.

**Table S1.** Clinical pathological characteristics of SCCHN patients’ tissue microarray.
