# Correction to ‘TRAF6 Regulates Tumour Metastasis Through EMT and CSC Phenotypes in Head and Neck Squamous Cell Carcinoma’

**DOI:** 10.1111/jcmm.70750

**Published:** 2025-08-13

Chen L, Li YC, Wu L, Yu GT, Zhang WF, Huang CF, Sun ZJ. TRAF6 regulates tumour metastasis through EMT and CSC phenotypes in head and neck squamous cell carcinoma. *Journal of Cellular and Molecular Medicine* 2017; 22: 1337–1349. https://doi.org/10.1111/jcmm.13439

In Lei Chen et al., Figure 1, 2, 3, and 4 contained errors. The hematoxylin‐eosin (HE) image of lymph node metastasis in Figure 1C, the Western blot images in Figures 2A, 3B and 4B and the transwell assay image of the negative control (NC) group in Figure 2F are incorrect. The correct images are shown below. These corrections do not alter the results or conclusions of the article. All authors have reviewed and approved these corrections.

**FIGURE 1C:**
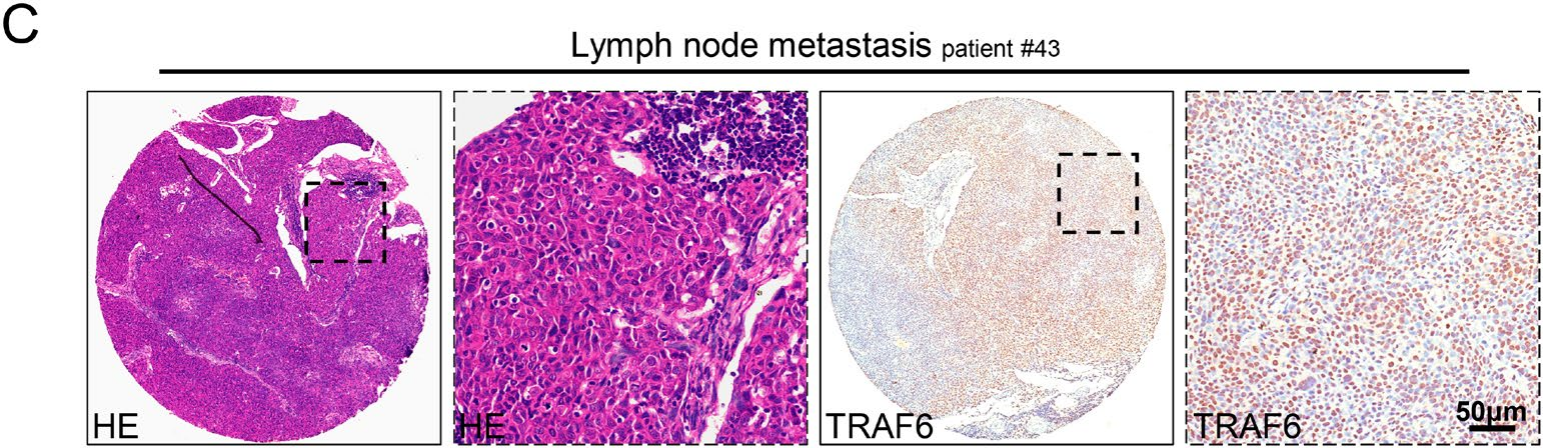
Elevated expression of TRAF6 was closely related to lymph node status in human SCCHN tissue. (C) Representative HE and IHC images of TRAF6 in metastatic lymph node. Scale bars = 50 μm.

**FIGURE 2A and F:**
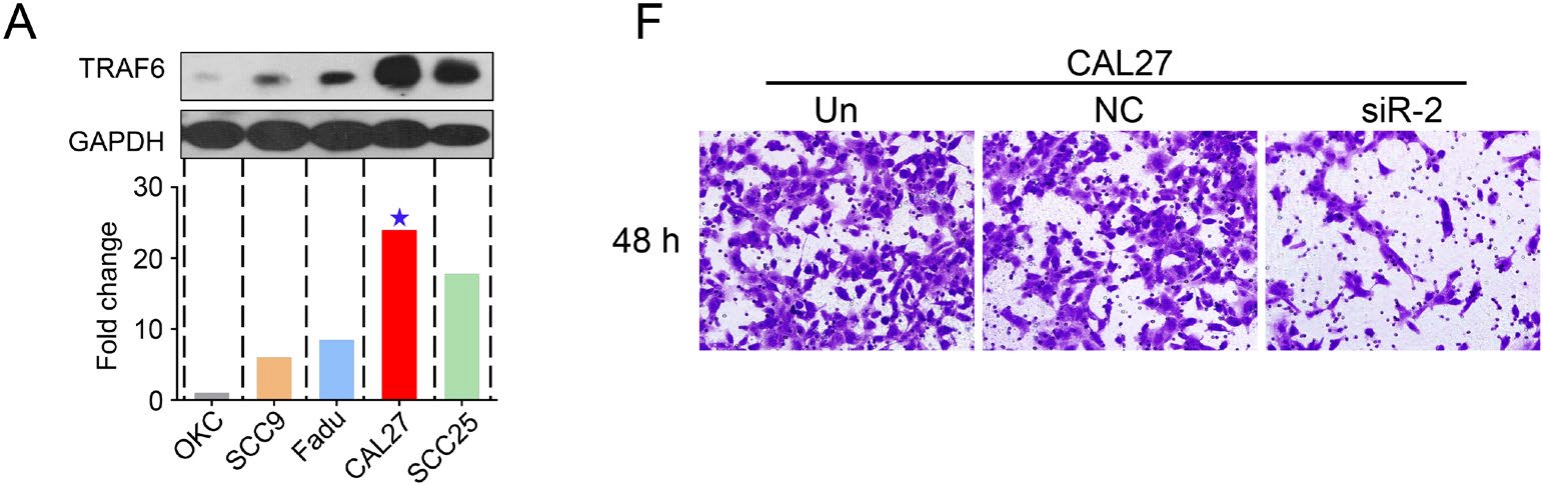
The EMT process, invasiveness and metastasis ability were inhibited by knocking down TRAF6 in SCCHN cells. (A) Western blot analysis was performed to examine TRAF6 expression in several SCCHN cell lines and the OKC cell line. (F) Representative microphotographs of the transwell assay (magnification, 40×) of invaded cells. The photograph was taken 48 h after cell plating.

**FIGURE 3B:**
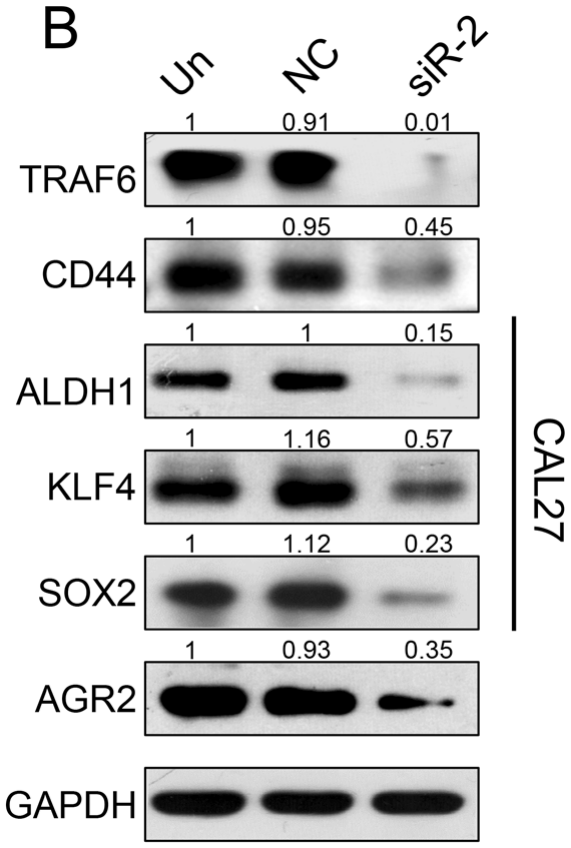
Effect of TRAF6 knockdown on cancer stem cell formation in SCCHN cell lines. (B) Western blot analysis showed that CD44, ALDH1, KLF4, SOX2 and AGR2 protein expressions were remarkably decreased after knocking down TRAF6 in the CAL27 cell line.

**FIGURE 4B:**
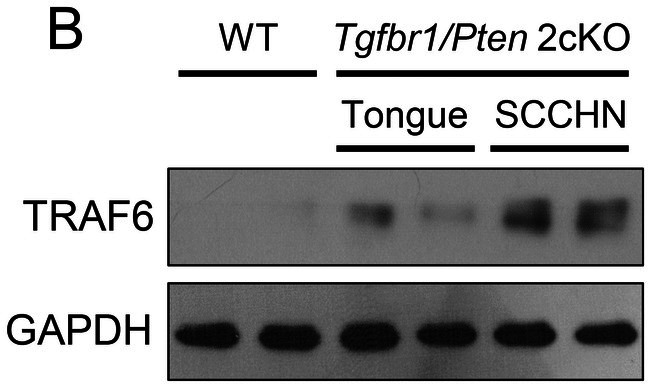
High expression of TRAF6 is correlated with CSC‐ and EMT‐related protein in *Tgfbr1*/*Pten* 2cKO mice. (B) Western blot analysis of TRAF6 expression in the tongue of wild‐type (WT) mice and in both the tongue and tumour tissues of *Tgfbr1*/*Pten* 2cKO mice.

We apologise for these errors.

