# Supplementary material for: TRAF6 regulates tumour metastasis through EMT and CSC phenotypes in head and neck squamous cell carcinoma
Source: J Cell Mol Med. 2017 Nov 29;22(2):1337–49. doi: 10.1111/jcmm.13439 (PMC5783876; doi:10.1111/jcmm.13439)
Supplement: Supplementary file 4 — Table S1. Clinical pathological characteristics of SCCHN patients’ tissue microarray. [file JCMM-22-1337-s004.docx]

**Supplementary Table S1**

Clinical pathological characteristics of SCCHN patients

| ***Case*** | ***Patients*** | ***Type*** | ***Sex*** | ***Age*** | ***Location*** | ***TNM*** | ***Grade*** |
| --- | --- | --- | --- | --- | --- | --- | --- |
| 1 | 1 | HNSCC | Male | 67 | Tongue | T2N0M0 | I |
| 2 | 2 | HNSCC | Male | 55 | Retromolar Pad | T1N1M0 | I |
| 3 | 3 | HNSCC | Male | 38 | Tongue | T1N1M0 | I |
| 4 | 4 | HNSCC | Male | 75 | Tongue | T2N0M0 | I |
| 5 | 5 | HNSCC | Male | 55 | Gingvia | T2N0M0 | I |
| 6 | 6 | HNSCC | Male | 55 | Tongue | T1N0M0 | I |
| 7 | 7 | HNSCC | Female | 74 | Tongue | T2N1M0 | I |
| 8 | 8 | HNSCC | Female | 60 | Tongue | T1N0M0 | I |
| 9 | 9 | HNSCC | Male | 42 | Gingvia | T2N0M0 | I |
| 10 | 10 | HNSCC | Male | 59 | Buccal mucosa | T2N1M0 | I |
| 11 | 11 | HNSCC | Female | 65 | Buccal mucosa | T2N0M0 | II |
| 12 | 12 | HNSCC | Male | 40 | Buccal mucosa | T3N1M0 | II |
| 13 | 13 | HNSCC | Male | 38 | Buccal mucosa | T3N0M0 | II |
| 14 | 14 | HNSCC | Male | 53 | Tongue | T2N1M0 | II |
| 15 | 15 | HNSCC | Male | 67 | Tongue | T3N0M0 | II |
| 16 | 16 | HNSCC | Female | 63 | Tongue | T3N0M0 | II |
| 17 | 17 | HNSCC | Male | 59 | Tongue | T2N0M0 | II |
| 18 | 18 | HNSCC | Male | 49 | Tongue | T2N1M0 | II |
| 19 | 18 | LN |  |  |  |  |  |
| 20 | 19 | HNSCC | Male | 70 | Tongue | T1N1M0 | II |
| 21 | 20 | HNSCC | Male | 88 | Tongue | T2N1M0 | II |
| 22 | 21 | HNSCC | Male | 72 | Tongue | T2N1M0 | II |
| 23 | 22 | HNSCC | Male | 73 | Tongue | T2N0M0 | II |
| 24 | 23 | HNSCC | Male | 41 | Tongue | T3N0M0 | II |
| 25 | 24 | HNSCC | Male | 54 | Tongue | T2N1M0 | II |
| 26 | 24 | LN |  |  |  |  |  |
| 27 | 25 | HNSCC | Male | 63 | Soft Palate | T2N0M0 | II |
| 28 | 26 | HNSCC | Male | 38 | Tongue | T2N0M0 | II |
| 29 | 27 | HNSCC | Male | 42 | Gingvia | T2N0M0 | III |
| 30 | 28 | HNSCC | Male | 65 | Buccal mucosa | T3N1M0 | III |
| 31 | 29 | HNSCC | Male | 67 | Tongue | T2N0M0 | III |
| 32 | 30 | HNSCC | Male | 79 | Buccal mucosa | T2N1M0 | III |
| 33 | 31 | HNSCC | Male | 49 | Gingvia | T2N0M0 | III |
| 34 | 32 | HNSCC | Male | 53 | Retromolar Pad | T2N0M0 | III |
| 35 | 33 | HNSCC | Male | 55 | Tongue | T3N1M0 | III |
| 36 | 34 | HNSCC | Male | 69 | Mouth floor | T2N1M0 | III |
| 37 | 65 | HNSCC | Male | 64 | Tongue | T3N1M0 | III |
| 38 | 66 | HNSCC | Male | 60 | Tongue | T3N0M0 | III |
| 39 | 67 | HNSCC | Male | 49 | Tongue | T3N0M0 | III |
| 40 | 68 | HNSCC | Female | 55 | Tongue | T2N0M0 | Ⅰ |
| 41 | 39 | HNSCC | Male | 49 | Tongue | T2N0M0 | II |
| 42 | 40 | HNSCC | Female | 53 | Buccal mucosa | T1N0M0 | II |
| 43 | 41 | HNSCC | Female | 75 | Buccal mucosa | T1N0M0 | II |
| 44 | 42 | HNSCC | Female | 73 | Tongue | T2N1M0 | II |
| 45 | 43 | HNSCC | Female | 65 | Tongue | T2N2M0 | II |
| 46 | 43 | LN |  |  |  |  |  |
| 47 | 69 | HNSCC | Male | 69 | Retromolar Pad | T2N0M0 | II |
| 48 | 70 | HNSCC | Male | 58 | Gingvia | T2N0M0 | II |
| 49 | 71 | HNSCC | Male | 39 | Tongue | T1N0M0 | II |
| 50 | 72 | HNSCC | Male | 54 | Mouth floor | T2N0M0 | II |
| 51 | 73 | HNSCC | Male | 71 | Retromolar Pad | T3N0M0 | II |
| 52 | 74 | HNSCC | Female | 57 | Retromolar Pad | T2N1M0 | II |
| 53 | 75 | HNSCC | Male | 42 | Buccal mucosa | T2N0M0 | II |
| 54 | 76 | HNSCC | Male | 43 | Palate | T2N0M0 | II |
| 55 | 52 | HNSCC | Male | 54 | Mouth floor | T1N0M0 | II |
| 56 | 53 | HNSCC | Male | 55 | Mouth floor | T2N1M0 | II |
| 57 | 53 | LN |  |  |  |  |  |
| 58 | 54 | HNSCC | Male | 60 | Retromolar Pad | T2N0M0 | II |
| 59 | 55 | HNSCC | Male | 63 | Tongue | T2N0M0 | II |
| 60 | 56 | HNSCC | Male | 71 | Retromolar Pad | T2N0M0 | II |
| 61 | 56 | LN |  |  |  |  |  |
| 62 | 57 | HNSCC | Male | 70 | Tongue | T2N1M0 | Ⅲ |
| 63 | 58 | HNSCC | Male | 71 | Mouth floor | T2N0M0 | Ⅲ |
| 64 | 59 | HNSCC | Male | 63 | Tongue | T2N0M0 | Ⅲ |
| 65 | 77 | HNSCC | Male | 71 | Palate | T2N0M0 | Ⅲ |
| 66 | 78 | HNSCC | Female | 70 | Tongue | T1N0M0 | Ⅲ |
| 67 | 79 | HNSCC | Female | 51 | Buccal mucosa | T2N1M0 | Ⅲ |
| 68 | 80 | HNSCC | Male | 78 | Underlip | T1N0M0 | Ⅲ |
| 69 | 81 | HNSCC | Male | 53 | Tongue | T2N0M0 | Ⅲ |
